# Perinatal Catatonia in a Patient with a Twin Pregnancy of Unknown Chorionicity and Gestational Age Presenting in Spontaneous Preterm Labor

**DOI:** 10.1155/2022/3143601

**Published:** 2022-04-12

**Authors:** Ryan Farias, Janice Hartnett

**Affiliations:** University of Connecticut Obstetrics and Gynecology, USA

## Abstract

Catatonia during pregnancy is rare and presents unique challenges due to the potential ramifications to mom and baby of the overall disease state and of potential treatment options. The purpose of this case report is to highlight the complexities in the workup and management of a catatonic patient with concurrent acute obstetric concerns requiring urgent intervention. We report a case of acute catatonia due to underlying major depressive disorder in a patient who presented in spontaneous preterm labor, with a twin pregnancy of unknown chorionicity with no known prenatal care. She underwent an extensive workup with no significant findings on lumbar puncture, brain MRI, metabolic labs, and EEG. After exclusion of several acute underlying conditions, a presumptive diagnosis of catatonia secondary to exacerbation of underlying major depressive disorder was made. She was transferred to an inpatient psychiatric facility postdelivery and treated with a course of lorazepam, aripiprazole, and escitalopram with good effect.

## 1. Introduction

Catatonia is a neuropsychiatric behavioral syndrome characterized by an inability to move normally, abnormal behaviors, and withdrawal. Clinical manifestations encompass a heterogeneous group of signs/symptoms including mutism, stupor, atypical posturing, waxy flexibility, and staring. There are a wide variety of potential underlying etiologies, including bipolar disorder, major depressive disorder, schizophrenia, meningitis, autoimmune encephalitis, neoplasm, or various other infectious, metabolic, neurologic, and rheumatologic disorders, which can make diagnosis and management difficult. During pregnancy, catatonia is more commonly due to an underlying organic process such as neuroleptic malignant syndrome, paraneoplastic encephalitis, atypical posterior reversible encephalopathy, eclampsia, and cerebrovascular disease. Catatonia during pregnancy is rare and presents unique challenges due to the potential ramifications to mom and baby of the overall disease state and of potential treatment options. We report a case of catatonia due to underlying major depressive disorder in a pregnant patient who presented to labor and delivery with a spontaneous twin pregnancy of unknown chorionicity, with no prenatal care, at an unknown gestational age.

## 2. Case

A 36-year-old G6P5005 with no prenatal care and a spontaneous twin pregnancy of unknown chorionicity presented with altered mental status. She was accompanied by her partner, and he reported that she was “two days past her due date” and was becoming “more unresponsive.” The patient was not able to speak any words and would respond to questions only with simple noises. She was able to point to the words “yes” and “no” written on paper to answer certain questions and was able to express that she felt safe with her partner and did not feel as if she was in any danger. The majority of the patient history was obtained from the patient's partner, who denied any history of psychiatric illness or hospitalization. Limited bedside growth scan was consistent with approximately 36-week gestational age, and both babies were in breech presentation. The psychiatry team was consulted, and, on their evaluation, they noted that she required multiple verbal prompts to follow simple commands and demonstrated reduced response to stimulation over time with progressive difficulty moving her fingers to point to “yes/no” written on paper. On mental status exam, she was noted to have poor eye contact, psychomotor slowing, and flat affect and appeared to be internally occupied. She pointed to “yes” when asked about hearing voices. Given the constellation of mutism, difficulty moving, waxy flexibility on prompting, fluctuating alertness, and internal preoccupation, a diagnosis of hypoactive catatonia was made. She was started on lorazepam 1 mg every two hours.

**Figure 1:**
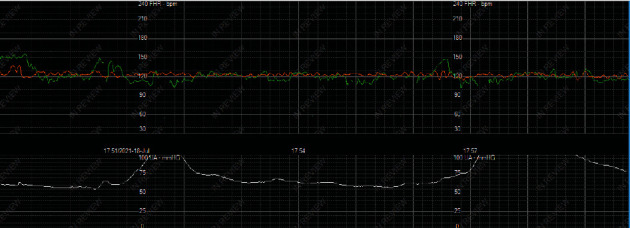
Fetal cardiotocography tracing on admission to labor and delivery.

From an obstetric standpoint, she was also noted to have an irritable contraction pattern on tocometry and, after consenting to a cervical exam, was found to be 4 cm dilated/50% effaced/-3 station. Fetal cardiotocography from time of admission is shown in Figure 1. She became very combative during attempts at IV placement and was unable to be put in wrist restraints to safely obtain IV access. The case was discussed with the hospital Risk Management team and Maternal-Fetal Medicine. Per Risk Management, the team should care for the patient as medically necessary, including the use of medical sedation and physical restraints, if needed. She was given 5 mg intramuscular haloperidol and 2 mg intramuscular lorazepam for sedation which allowed for safe placement of an IV. She continued to regularly contract and on repeat exam was found to be 5-6 cm dilated/75% effaced/-2 station with breech-breech presentation reconfirmed on ultrasound. Per prior psychiatry evaluation, the patient did not have capacity to give informed consent and would be unable to consent for primary cesarean section for spontaneous preterm labor with fetal malpresentation. She was able to respond “okay” when informed of need to proceed with surgery but was unable to sign consent. She underwent an uncomplicated primary cesarean section under general anesthesia.

The Psychiatry team continued to follow postoperatively, and she was transitioned to Lorazepam 2 mg every six hours, which slowly began to produce a clinical response. On postop day 0, she was noted to be intermittently verbal with more purposeful movements and was able to answer questions at times. On postop day 1, she was able to ambulate with supervision, eat and use the bathroom on her own, and visit her babies in the NICU. However, her overall mental status would wax and wane throughout the day, and she continued to have nonverbal periods with psychomotor slowing. It was determined that she met criteria for inpatient psychiatric hospitalization given her grave disability secondary to her catatonic state. She was transitioned to lorazepam 2 mg every 8 hours and underwent lumbar puncture to rule out autoimmune encephalitis prior to being discharged to the nearby psychiatric hospital on postop day 3. Her workup prior to discharge to the psychiatric hospital included normal LP, metabolic labs, and no evidence of seizure on EEG. Brain MRI showed artifact versus reduced diffusivity within the caudate nuclei bilaterally and the cerebral cortex bilaterally, which can frequently be seen in acute catatonia. The diagnostic findings from the case are summarized in Table 1. Prior to her discharge, records from a hospital in a different state were obtained which showed a long-standing history of major depressive disorder with catatonic features, for which she had previously been prescribed lorazepam 1 mg three times daily. During her psychiatric admission, lorazepam was uptitrated to 3 mg three times daily due to continued persistent catatonia and frequent relapses. The lorazepam treatment had a waxing and waning effect on her catatonic features, showing baseline functionality after her dosages that regressed back to catatonic before the next dose was due. She was also started on aripiprazole due to possible psychosis with reported auditory hallucinations, as well as escitalopram due to depressed mood and a previous diagnosis of major depressive disorder. ECT was offered several times during the patient's admission, but each time, the patient declined. She was eventually discharged to the care of her father after a 33-day stay with improved mood and a Bush-Francis score of 0 with continued lorazepam treatment. She was discharged on lorazepam, aripiprazole, and escitalopram, with plan for close outpatient follow-up.

## 3. Discussion

Catatonia generally occurs in patients with an underlying psychiatric or general medical disorder such as bipolar disorder, major depressive disorder, schizophrenia, meningitis, autoimmune encephalitis, neoplasm, or various other infectious, metabolic, neurologic, and rheumatologic disorders. There are three main subtypes of catatonia, hypoactive, excited, and malignant. Hypoactive, which is what our patient displayed, is characterized by mutism, inhibited movement, negativism, and staring. Patients with excited catatonia exhibit excessive and purposeless motor activity in their extremities with restlessness, impulsivity, and combativeness. Finally, malignant catatonia is a life-threatening state that is accompanied by fever, autonomic instability, delirium, and rigidity. These subtypes exist on a spectrum, and patients may transition back-and-forth at any given time. The most widely used scoring system in the evaluation and tracking of treatment over time in catatonic patients is the Bush-Francis Catatonia Rating Scale, which is a 23-item rating scale with good interrater reliability.

Overall, catatonia is rare in the peripartum period. A search of the National Library of Medicine database by Gonzales et al. discovered 16 reported cases of catatonia occurring during pregnancy or in the immediate postpartum period [1]. Many of these cases were due to organic medical conditions including NMS, paraneoplastic encephalitis, atypical posterior reversible encephalopathy, eclampsia, and cerebrovascular disease. Thus, when any pregnant patient presents with catatonic features, it is important to keep a broad differential and rule out underlying medical conditions that could potentially be fatal to mom and/or baby. The first-line treatment of catatonia is high-dose benzodiazepines, which also aids in making the diagnosis. The lorazepam challenge involves administering an IV bolus of 1 to 2 mg and closely observing for improvement in symptoms. Partial relief of signs/symptoms 5 to 10 minutes after administration is consistent with a diagnosis of catatonia [2, 3]. Treatment then involves a continued regimen of high-dose benzodiazepines, frequently followed by electroconvulsive therapy. Multiple prospective open-label studies, cases series, and retrospective studies suggest that benzodiazepines alone, ECT alone, or benzodiazepines followed by ECT lead to recovery in approximately 60 to 80 percent of patients [4]. Treatment guidelines from the American Psychiatric Association recommend treating catatonia with either a benzodiazepine or ECT, depending upon the clinical scenario [5].

The case presented demonstrates several of the unique challenges of catatonia during pregnancy. The immobility, mutism, and withdrawal of hypoactive catatonia, especially when combined with other socioeconomic factors, can make it very difficult for patients to receive adequate prenatal care. Our patient presented to the hospital with no available records, with a twin pregnancy of unknown chorionicity, at an unknown gestational age. Her partner was also unable to provide any reliable collateral information, which raised concern for domestic violence, unstable housing, and/or sex trafficking. Furthermore, she presented in spontaneous labor with a twin pregnancy with breech-breech presentation necessitating cesarean delivery. The patient's presentation made this case unique from prior reported cases of catatonia during pregnancy, as our patient presented both with acute catatonia and an acute obstetric concern necessitating delivery. It also presented a very ethically challenging situation as her partner was unavailable, and she was deemed to not have capacity to make medical decisions or provide informed consent. In this scenario, there is an ethical obligation to determine an alternative decision maker, which is ideally someone chosen by the patient in advance in the form of a spouse, child, sibling, or other close relative. Our patient had no immediately available alternative decision maker, and a prompt decision was needed given the acute change in clinical status and need for cesarean section.

An additional challenge of catatonia during pregnancy is the potential effects of the standard treatment options on the pregnancy. A 2011 meta-analysis of nine studies with more than one million pregnancies found that in general, benzodiazepines did not appear to increase teratogenic risk [6]. However, retrospective case-control studies suggest that fetal exposure to benzodiazepines may increase the risk of oral cleft from the general population base rate of 6 in 10,000 births to 11 in 10,000 births [7]. Furthermore, benzodiazepine use in the third trimester can lead to neonatal abstinence syndrome, temperature dysregulation, hypotonia, and poor feeding at birth [8]. Antenatal benzodiazepine use may also be associated with preterm birth and low birth weight [8]. In our case, benzodiazepine therapy was started very shortly prior to delivery, likely negating any possible negative effects. Postdelivery in breastfeeding moms, lorazepam has low levels in breastmilk and does not cause any adverse effects in breastfed infants [9]. The other mainstay of treatment of catatonia, electroconvulsive therapy, is largely considered to be safe at any stage of pregnancy. However, there have been case reports of placental abruption, preterm labor, miscarriage, and fetal heart rate decelerations associated with ECT [10]. Although not an issue in this case, as our patient had an urgent obstetric indication for delivery, the decision of when to deliver and route of delivery can be very difficult in catatonic patients. In general, decision making will depend on gestational age, severity of disease, other underlying medical conditions, and a risk-benefit analysis of intrapartum treatment vs. delay until after delivery.

This case also demonstrates the importance of a complete workup to determine the underlying cause of catatonia. Our patient underwent bloodwork to assess for any metabolic derangements, evaluation for preeclampsia, brain MRI to assess for structural abnormality, EEG to assess for seizures, and a lumbar puncture to assess for autoimmune encephalitis. With continued work to obtain further collateral information, we were able to attain records that endorsed a history of catatonia secondary to major depressive disorder, which aided in evaluation and allowed for a more definitive diagnosis. This case also highlights the benefits of having a multidisciplinary care team, which included general Obstetrics and Gynecology, Maternal-Fetal Medicine, Psychiatry, Neurology, Social Work, and Risk Management.

## Figures and Tables

**Table 1:** Summary of diagnostic findings.

MRI brain	“Artifact versus reduced diffusivity within the caudate nuclei bilaterally and the cerebral cortex bilaterally that can be correlated with underlying encephalitis, hypoxic ischemic changes, and/or toxic/metabolic derangements”
EEG	“Essentially normal natural sleep inpatient bedside routine EEG for age during awake and drowsy. Excessive diffuse beta activity was noted likely due to benzodiazepine use”
Lumbar puncture	Negative paraneoplastic autoantibody evaluation
Vitamin D	10 ng/mL (NL = 30-100 ng/mL)
Vitamin B12	165 pg/mL (NL = 243-894 pg/mL)
Folate	4.2 ng/mL (NL ≥ 7.2 ng/mL)
Complete blood count	Unremarkable
Complete metabolic panel	Unremarkable
Ammonia level	14 mmol/L (NL = 11-51 mmol/L)
Thyroid stimulating hormone level	1.62 mIU/L (NL = 0.27-4.20 mIU/L)
Urine toxicology screen	Negative

## References

[1] Gonzales N., Quinn D. K., Rayburn W. (2014). Perinatal catatonia: a case report and literature review. *Psychosomatics*.

[2] Fink M., Taylor M. A. (2009). *Catatonia: A Clinician's Guide to Diagnosis and Treatment*.

[3] Fink M., Taylor M. A. (2001). The many varieties of catatonia. *European Archives of Psychiatry and Clinical Neuroscience*.

[4] Coffey J., Roy-Byrne P., Marder S., Solomon D. (2021). *Catatonia: treatment and prognosis. UpToDate*.

[5] Gelenberg A. J., Freeman M. P., Markowitz J. C. (2010). American Psychiatric Association practice guidelines for the treatment of patients with major depressive disorder. *American Journal of Psychiatry*.

[6] Enato E., Moretti M., Koren G. (2011). Motherisk rounds: the fetal safety of benzodiazepines: an updated meta- analysis. *Journal of Obstetrics and Gynaecology Canada*.

[7] Yonkers K. A., Wisner K. L., Stowe Z. (2004). Management of bipolar disorder during pregnancy and the postpartum period. *The American Journal of Psychiatry*.

[8] Wikner B. N., Stiller C. O., Bergman U., Asker C., Källén B. (2007). Use of benzodiazepines and benzodiazepine receptor agonists during pregnancy: neonatal outcome and congenital malformations. *Pharmacoepidemiology and Drug Safety*.

[9] Drugs and Lactation Database (LactMed) (2006). *Lorazepam*.

[10] Saatcioglu O., Tomruk N. B. (2011). The use of electroconvulsive therapy in pregnancy: a review. *The Israel Journal of Psychiatry and Related Sciences*.

